# Global Impact of 60 years of *ES* *&T*

**DOI:** 10.1021/acs.est.5c17819

**Published:** 2026-01-08

**Authors:** Gregory V. Lowry, Zhiyong Jason Ren, Pedro J.J. Alvarez, Juliane Hollender, Shuxiao Wang, Margaret Mills, Julie B. Zimmerman

**Affiliations:** 1 Civil & Environmental Engineering, 6612 Carnegie Mellon University, Pittsburgh, Pennsylvania 15217, United States; 2 Department of Civil and Environmental Engineering, 6740 Princeton University, Princeton, New Jersey 08544, United States; 3 Department of Civil and Environmental Engineering, 3990 Rice University, Houston, Texas 77005, United States; 4 Eawag: Swiss Federal Institute of Aquatic Science and Technology, Ueberlandstrasse 133, Duebendorf 8600, Switzerland; 5 State Key Joint Laboratory of Environmental Simulation and Pollution Control, School of Environment, 12442 Tsinghua University, Beijing 100084, China; 6 American Chemical Society, ACS International Ltd., Binsey House, Wallbrook Court, Washington 20036-4892, United Kingdom; 7 Yale School of the Environment, Yale University, New Haven, Connecticut 06520, United States

**Keywords:** science impact, science policy, environmental research, environmental sustainability, human health

## Abstract

For 60 years, the *Environmental Science* *& Technology* research community has helped to define the fields of environmental science and engineering. The research topics have evolved over time to respond to the most pressing societal needs, from treatment technologies and pollution control strategies to address severe environmental pollution, to pollution prevention and industrial ecology to help mitigate emissions, and to defining planetary boundaries for sustainability. Since *ES* *&T* launched in 1967, it has helped to create a robust global network of researchers, with researchers from 144 countries now contributing to address critical global environmental and human health challenges. Throughout its six decades, *ES* *&T* research has remained highly relevant to understanding, addressing, and advancing solutions to both current and emerging challenges and for developing science-based policies to protect the environment and human health. We are optimistic that the *ES* *&T* research community will continue to serve to help shape research and action toward a healthier, resilient, and sustainable planet for all of us in the next 60 years.

## Graphical abstract

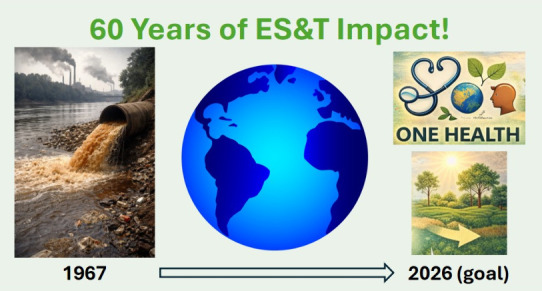

## Introduction

The global impact of the *Environmental Science* *& Technology* (*ES* *&T)* research community on the environment and human health over the past 60 years is undeniable. From its inception, the journal has not only assessed and improved the state of the environment and human health but also helped define the very fields of environmental science and engineering. Recognition of the mounting need to understand and restore the environment increasingly stressed by human activity (acid rain, algal blooms, unchecked industrial emissions of organic and inorganic pollutants and CO_2_), along with the tireless of efforts of many of the pioneers in the field, led to the inception of the ACS journal *Environmental Science* *& Technology* in 1967. The journal then nucleated a global community of researchers who could advance the needs of a burgeoning environmental movement and support a new era of environmental regulation and innovation with integrated knowledge and evidence-based solutions. Researchers publishing in *ES* *&T* have identified and raised awareness of critical environmental concerns and opportunities well before they emerged in public discourse. For example, the first decade of *ES* *&T* (1967–1977) was already raising concerns over the risks of disinfection byproducts formed in water treatment, the concept of bioavailability, and the need to be teaching “sustainability science”. *ES* *&T* research has always provided, and continues to provide, novel analytical methods, sampling procedures, foundational data sets, application-oriented laboratory experiments, and models that guide environmental monitoring and risk assessment informing technology development and policymaking.

Over six decades, the research topics published in *ES* *&T* have evolved alongside emerging chemicals, new planetary and human health risks, and tool and technology innovation. Yet the journal’s core mission has remained the same: “Scientific understanding of the environment and the development of chemical technologies for the environment are not ends in themselves. The goal is the benefit of [hu]­man”. The published research, reviews, policy analyses, perspectives, and viewpoints continue to identify new threats and shape the decisions of scientists, engineers, educators, citizens, and policymakers around the world.

## Emergence and Evolution of Environmental Research Topics over Six Decades

Broadly, environmental engineering and sciences were initially driven by anthropocentric ethics (e.g., public health protection), but more ecocentric drivers such as environmental and planetary health have become more prominent in recent decades. The nature and scale of the most pressing environmental problems have also changed significantly since the first Earth Day in 1970. While most cities have cleaner air and most lakes and rivers are more “fishable and swimmable”, largely due to improvements in point-source pollution control, we now face more intractable and less visible pollutants, more global and complex challenges that transcend international boundaries, and longer environmental effects and response times.

Using a broad brush, the predominant research activity in *Environmental Science & Technology* evolved with the pressing challenges of each decade (Figure 1) and resulted in a continuing decline of municipal and industrial emissions of pollutants (with CO_2_ being an exception to this rule). In the 1970s, the Clean Water Act stimulated research focused on municipal wastewater treatment and surface water pollution control. The 1980s experienced a significant increase in research on air quality and air pollution control. Hazardous waste management and soil and groundwater remediation were dominant topics in the 1990s. Research on more proactive approaches such as industrial ecology and green engineering became more prevalent at the turn of the 21st century, and climate change mitigation and planetary health have become a global research priority in the last few decades.

**Figure 1:**
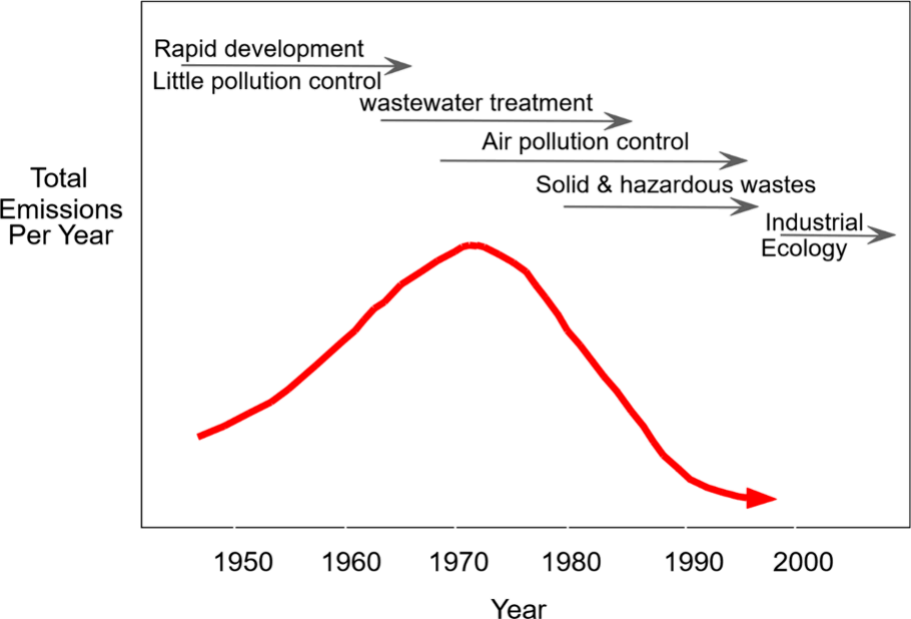
Evolution of predominant research activity in *Environmental Science & Technology* and the direction of change of anthropogenic emissions of contaminants of concern from the journal’s inception in 1967 to the 2000s (courtesy of Jerry Schnoor, ES&T EIC from 2003 to 2014).

A deeper dive into the topics of papers published in *ES* *&T* over the decades reflects this trend and highlights not only a sequence of research milestones but also a progressive broadening and deepening of the field itself (Figure 2). In the first decade of *ES* *&T*, the U.S. EPA had not yet been created and the U.S. Clean Air Act (1970) and U.S. Clean Water Act (1972) had not yet been enacted. Not surprisingly, most of the published content in *ES* *&T* during this decade (1967–1976) centered on characterizing organic and inorganic contaminants in air and water, supported by advances in sampling methods, analytical chemistry, and early air quality models. These foundational studies not only expanded scientific understanding but also directly informed the development and enforcement of landmark environmental regulations once they emerged, as well as the novel technologies necessary to comply with and surpass these regulatory limits. Many early themes were driven by the environmental crises of the time, e.g., managing acidification contributing to acid rain, controlling mercury pollution, and understanding the risks posed by large scale use of pesticides. Interestingly, the formation and implications of disinfection byproducts, the importance of contaminant bioavailability to risk, and sustainability science have been topics introduced during this decade. They were forward looking and important then, and those topics still persist today.

**Figure 2:**
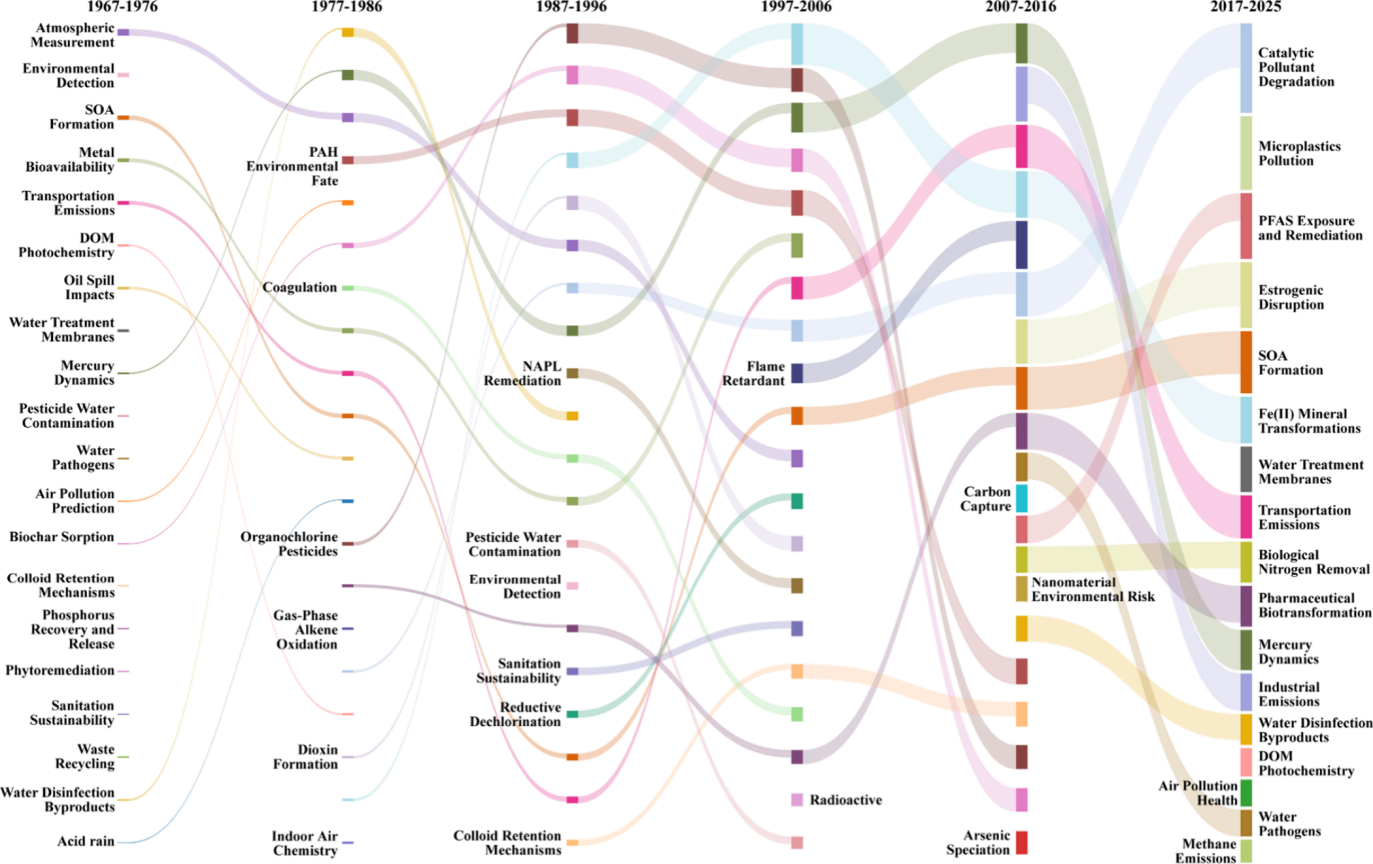
Thematic evolution of *ES* *&T* research over time, decade by decade popular topics, and overall trends.

An important section of *ES&T* was filled with magazine-style articles in the “Outlooks” and “Currents” section. This later became the “A-pages”. These sections provided recommendations about the governments’ roles in protecting natural resources and the environment, including articulating a clear path for implementing the 1967 Air Quality Act, for protecting waters from eutrophication, and for investments in both R&D spending and water infrastructure spending. These pages served as an important science communication tool to promote science-based policy decisions.

Research on many of these seminal and important environmental topics continued into the next decade (1977–1986), but critical new risks and solutions were identified. For example, the occurrence, fate, and risks of pharmaceuticals in wastewater and the formation and release of persistent organic pollutants, including dioxins, polychlorinated biphenyls (PCBs), and polycyclic aromatic hydrocarbons (PAHs), emerged as critical issues. These developments reflected a shift toward understanding how contaminants transform, persist, and interact within environmental matrices, enabling regulators to assess risks more accurately and innovators to develop new treatment approaches.

The third decade of *ES* *&T* (1987–1996) saw the rise of a movement toward novel treatment technologies for many of the pollutants in our air and water. It introduced novel approaches for addressing the impacts of chlorinated solvents and other persistent halogenated contaminants of concern, including novel pesticides and herbicides, in groundwater. It pioneered bioremediation strategies that were more passive, often employing natural attenuation, and were more sustainable than the existing resource-intensive technologies (pump and treat). This decade also advanced the understanding of colloid chemistry that enables more effective water treatment. These technologies and approaches eventually became widely adopted and accepted by regulators to help manage polluted water sources and remediate contaminated sites. This decade also introduced the concepts of “pollution prevention” and “waste reduction” as important environmental objectives, which are critical elements of the 1990 U.S. Pollution Prevention Act. Sustainability science such as environmental lifecycle assessment (LCA) was growing and helping to avoid pollution through, e.g., product substitutions with safer chemicals, rather than cleaning up toxic chemicals released to the air and water.

Crossing the new century (1997–2006) shed light onto new and emerging risks to and solutions for the environment and human health. There was significant work highlighting the occurrence, fate, and treatment of brominated flame retardants and elucidating the processes controlling the fate of uranium present at many large-scale military and industrial sites across the United States and the world. These studies developed many methods used today to manage uranium at these facilities. This decade also saw significant research on mercury methylation as the pathway to making it bioavailable and entering the food web. Deciphering these mechanisms helped management decisions to lower risks at mercury-contaminated sites. This decade also saw the rise of photocatalytic methods to degrade pollutants in water. The ability to use sunlight for treatments had the benefit of lowering the overall lifecycle costs associated with treatments, and novel photoactive materials are still being developed to manage persistent organic pollutants today (e.g., PFAS).

During this period, *ES&T* also became more international and opened editorial offices in Switzerland (Swiss Federal Institute of Aquatic Science and Technology, EAWAG) and China (Chinese Academy of Sciences Research Center for Eco-Environmental Sciences). This influence is reflected in the broadening of international research networks as discussed below.

The decade from 2007 to 2016 marked a period of rapid expansion in climate-and system-oriented environmental science, with *ES* *&T* emerging as a central venue for research addressing global change. A surge of studies examined greenhouse gas emissions, carbon capture, and carbon footprints of industrial processes, supported by the growing adoption of lifecycle assessment (LCA). In atmospheric science, *ES* *&T* published foundational work on secondary organic aerosols (SOA) and their important impacts on air quality, climate, and human health. This decade also highlighted the range of pharmaceuticals in waterways and their impacts, including estrogenic effects, leading to greater attention on endocrine disrupting chemicals more broadly. Next to chemical analysis, bioassays became more prominent to characterize mixtures in environmental samples including unknown chemicals and to replace in vivo experiments according to the 3R-principles (Replace, Reduce, Refine). Additionally, *ES* *&T* published many papers on the occurrence, fate, toxicity, and environmental implications of engineered nanomaterials, an emerging material class at that time and an emerging contaminant whose potential impacts were unknown. The evolution of knowledge about the potential environmental impacts of nanotechnology while the technology was just emerging in commerce developed the pursuit of “safe by design” approaches adapted from prior work on green chemicals.

The most recent papers in *ES* *&T* (2017–2025) reflect the period of accelerating environmental complexity and emerging opportunities. It is marked by pressing topics including the potential impacts of microplastics and PFAS exposures on the environment and human health as well as the application of data-driven AI and machine learning tools to better understand complex environmental systems and inform systems-level solutions. There is also a greater number of papers on air quality, with SOA and transportation emissions being critical topics as well as carbon capture, sequestration, and utilization. Interestingly, many important topics have remained relevant over 60 years at *ES* *&T*, such as the occurrence and management of disinfection byproducts, mercury dynamics, DOM photochemistry, Fe­(II) mineral transformations, water treatment membranes, and air pollution health impacts, highlighting the remaining importance of these topics in protecting human health and the environment. Together, these trends demonstrate a field confronting both emerging and persistent environmental challenges that have evolved from local to global and acute to chronic, requiring innovations in analytical and computational tools as well as technologies and policy mechanisms to address the challenges using inter- and transdisciplinary approaches.

## Evolution of an *Environmental Science & Technology* Research Community and Collaborative Research Networks

Environmental challenges are inherently global, and progress depends on the participation of a diverse set of stakeholders to find effective and equitable solutions. In response, the evolution of the *ES* *&T* author networks has grown to be more inclusive over the decades. Figure 3 shows the evolution of corresponding author institutions from the 1990s to 2020–2025.

**Figure 3:**
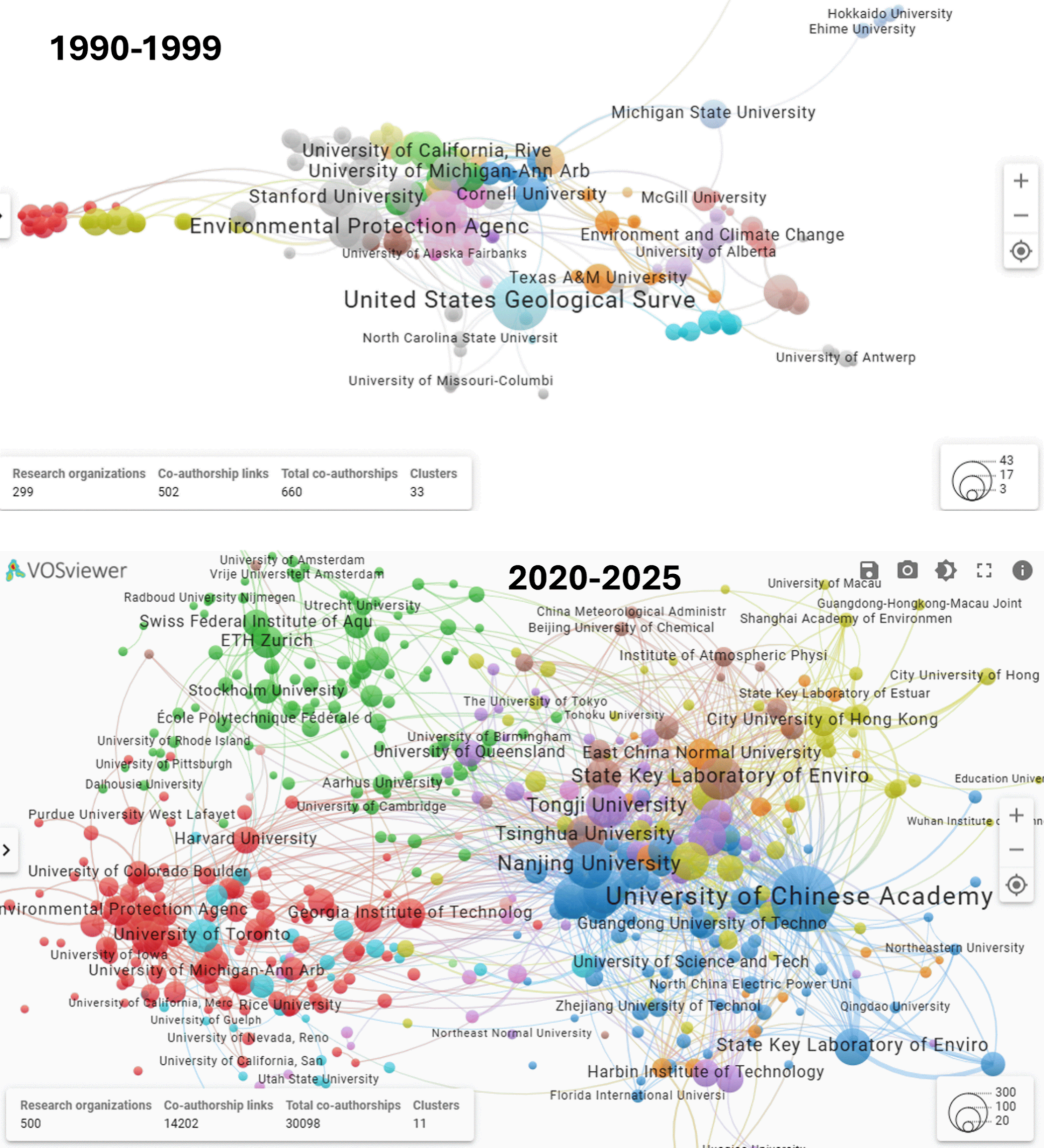
Evolution of *ES* *&T* author networks from the 1990s to the most recent five years (2020–2025). Researchers published in *ES* *&T* are now from 144 different countries. Source: Dimensions AI Database (https://www.dimensions.ai/).

Published work in the 1990s was clearly dominated by North American institutions, primarily in the United States. Both the United States Geological Survey (USGS) and the U.S. Environmental Protection Agency (U.S. EPA) had outsized contributions to the published content in *ES* *&T*, with strong representation from leading Canadian universities and Federal Agencies. During this period, both the USGS and U.S. EPA had significant research programs, and the agencies’ missions aligned well with the *ES* *&T* scope and purpose. There was also limited networking between the institutions in the 1990s. In the 2000s, with Federal grant support for large multi-investigator and multi-institution collaborations, the research network has expanded considerably, especially across North America and Europe, with early contributions from emerging research hubs in China. In the 1990s, the European framework programs became an important pillar for mission-oriented research with environmental relevance in Europe. Although authorship remained concentrated in traditional scientific powers, the decade marked the beginning of more frequent cross-border collaborations.

In addition to published research by the United States and the EU, the 2010s show a significant rise in *ES* *&T* published works from Chinese institutions. There were some highly productive institutions during that period, especially in Switzerland (ETH), the United States (U.S. EPA and National Laboratories), and the University of the Chinese Academy of Sciences. The connectivity between the different research institutions also continues to rise in the 2010s. This rise corresponds to a significant increase in environmental research in China, including infrastructure and research Centers. It also corresponds to the initiation of joint research projects between different countries. For example, the U.S. National Science Foundation (USNSF) and National Science Foundation of China (NSFC) issued its first joint call for collaborative projects in the mid-2010s. Similar programs between the United States and UK and the United States and EU were also occurring. The research network connectivity growth in the 2010s and even more in the 2020s is testament to the success of these important collaborative research funding mechanisms. In the 2020s, the major authors’ regions are largely the same, but the network of Chinese universities continues to expand, both within China institutions and between Chinese institutions and the rest of the world. Importantly, the network connection between all researchers in each network appears to be expanding. Six decades after its initiation, *ES* *&T* now represents work from researchers from 144 countries (Figure 4).

**Figure 4:**
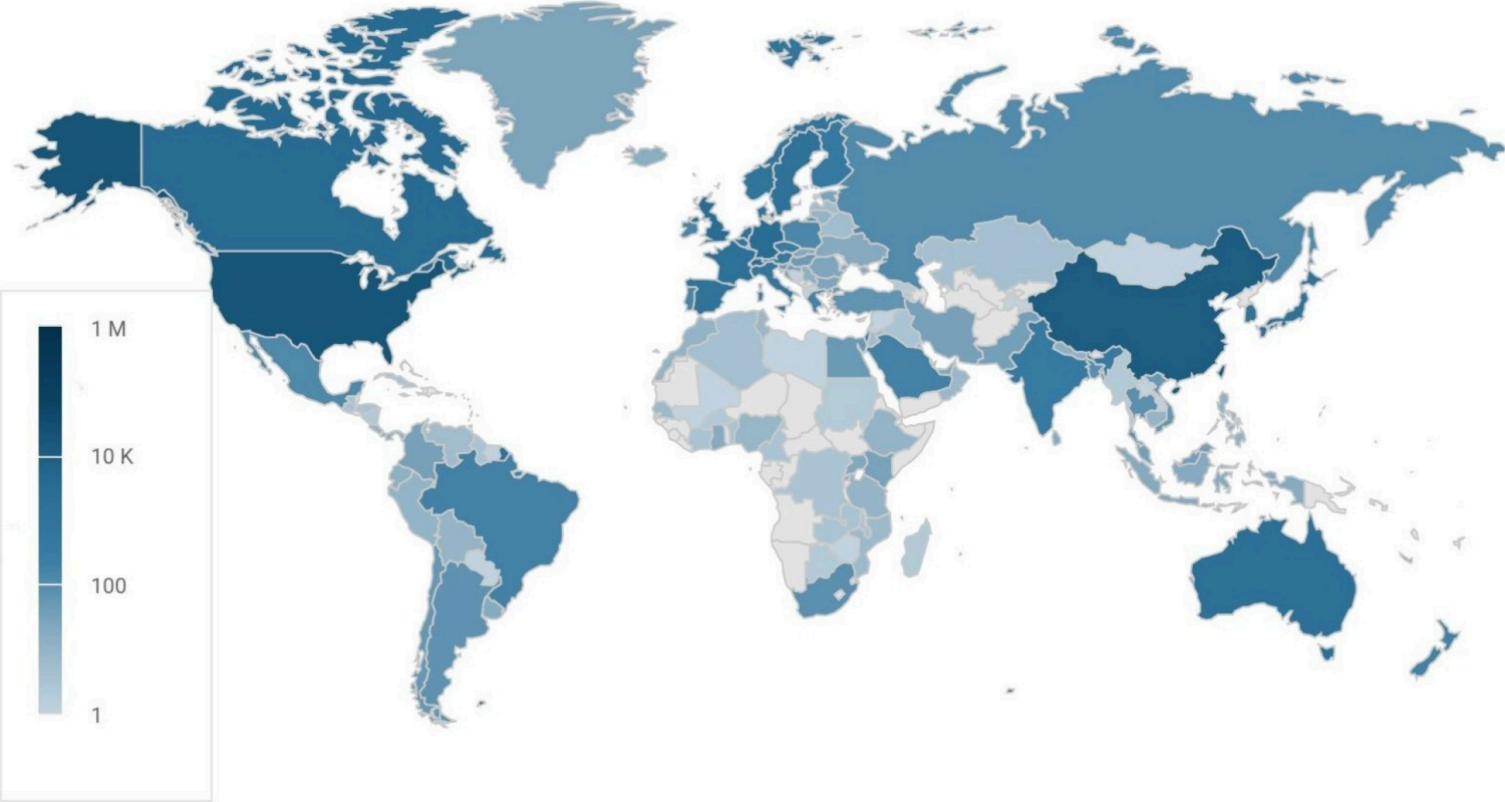
There have been published manuscripts with the corresponding author institution in 144 countries since 1967. The scale bar indicates the number of published papers. For perspective on volume, the blue color of the USA corresponds to 19,400 published manuscripts. Source: Dimensions AI Database (https://www.dimensions.ai/).

The more recent challenging environmental problems such as the ubiquity of PFAS and microplastics, as well as the impact of air pollution on human health, remain global concerns that require input from global stakeholders to advance solutions. This is nicely reflected in both the types of research being published in *ES* *&T* (Figure 2) and the network of researchers and stakeholders collaborating on these topics (Figure 3). Scientists and engineers are essential ambassadors of cooperation on global environmental topics like these. Despite this large increase in the *ES* *&T* research network over time, there is still work to do. For example, researchers from the Global South are still underrepresented in published research in *ES* *&T*. Plans are underway to continue to expand the global network of researchers addressing the most pressing global environmental and human health problems through regional engagement and special issues on relevant topics.

## Evolution of Environmental Journals since *ES* *&T* Was Launched in 1967

*ES* *&T* published content has had an enormous impact on research in the fields of environmental science, technology, and health. As one of the first peer-reviewed comprehensive environmental journals (i.e., it covers a wide range of environmental and human health topics) in 1967, it has encouraged a growing community of comprehensive environmental journals (e.g., *Science of the Total Environment*, *Environmental Toxicology* *& Chemistry*, *Journal of Hazardous Materials*, *Environment International*), as well as many more somewhat narrowly focused environmental journals including a number of its own family (*ACS ES* *&T Water*, *ACS ES* *&T Air*, *ACS ES* *&T Engineering*). The circular network shown in Figure 5 illustrates the evolution of the broader environmental publishing landscape. As one of the first, *ES* *&T* sits at the center of a rapidly expanding constellation of environmental journals, with citation links radiating outward over time as the number, diversity, and global reach of journals grow.

**Figure 5:**
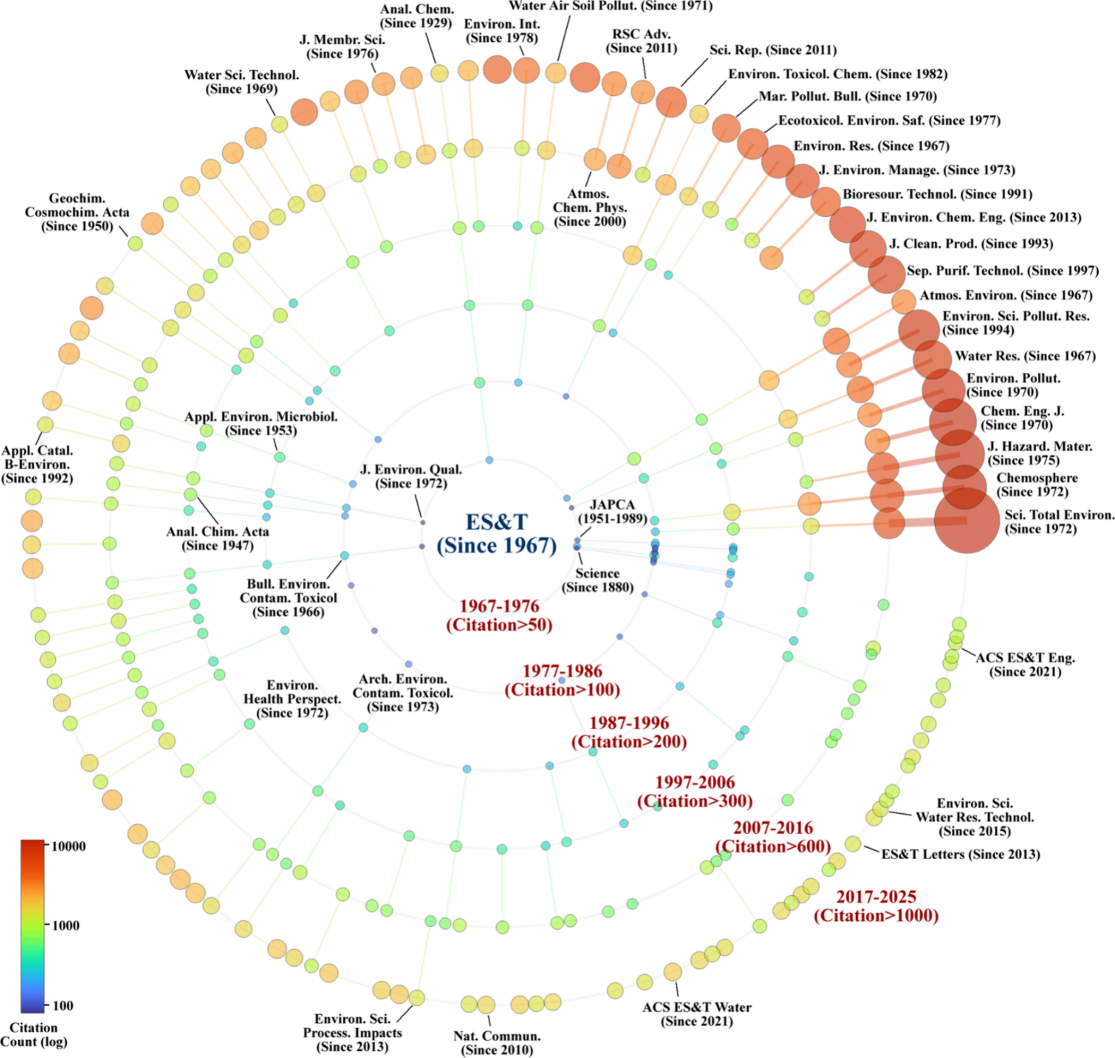
Selected broad spectrum environmental science and health journals launched since *ES* *&T* began in 1967. Each concentric circle represents a decade. The circles show the number of *ES* *&T* papers that have been cited in those journals for each decade.

*ES* *&T* has always strived to publish cutting-edge impactful science and policy analyses that can provide the foundational knowledge necessary to move the field forward in selected areas. These foundational papers have led to lines of inquiry on new topics and provided critical data sets that inform the research, education, and policymaking communities about relevant environmental concentrations or critical fate processes affecting them. For example, a study by USGS researchers in 2002 showing the prevalence of pharmaceuticals, hormones, and organic chemicals present in pristine water bodies across the United States^1^ has been cited over 11,000 times and was instrumental in illuminating the need for additional research toward this important environmental problem that still receives regular attention by the *ES* *&T* community today. Figure 5 shows that this large community of environmental journals is citing foundational *ES* *&T* studies at a high rate, with significant increases coming in the past two decades. The increasing citation density in these outer rings highlights *ES* *&T*’s sustained influence across the global environmental research community, as emerging journals draw on *ES* *&T*’s foundational literature to define new subfields. However, beyond growth, there is also increasing interconnectedness, as the journals across continents and disciplines increasingly cite one another, reflecting the rising complexity and inter- and transdisciplinarity of environmental science, technology, health, and policy.

## Impact on Environmental and Human Health Policy

*ES* *&T* research has had significant policy impacts both regionally and globally. A Dimensions AI database analysis (https://www.dimensions.ai/) estimates that *ES* *&T* papers have been used over 24,000 times in various policy documents by ∼250 different agencies in the USA (87) and internationally (162). The U.S. Center for Disease Control (CDC) cites *ES* *&T* papers the most, with over 3200 citations. This is followed by the United Nations Environment Programme (UNEP) (2247), National Academies Press (1960), and the World Health Organization (WHO) (1587). Other agencies significantly relying on *ES* *&T* science for policy development include the Washington State Department of Ecology, the Food and Agriculture Organization (FAO, United Nations), the Publications Office of the European Union, the Organisation for Economic Co-operation and Development (OECD), the U.S. EPA, and the Rijksinstituut voor Volksgezondheid en Milieu (RIVM, Netherlands National Institute for Public Health and the Environment) (Figure 6).

**Figure 6:**
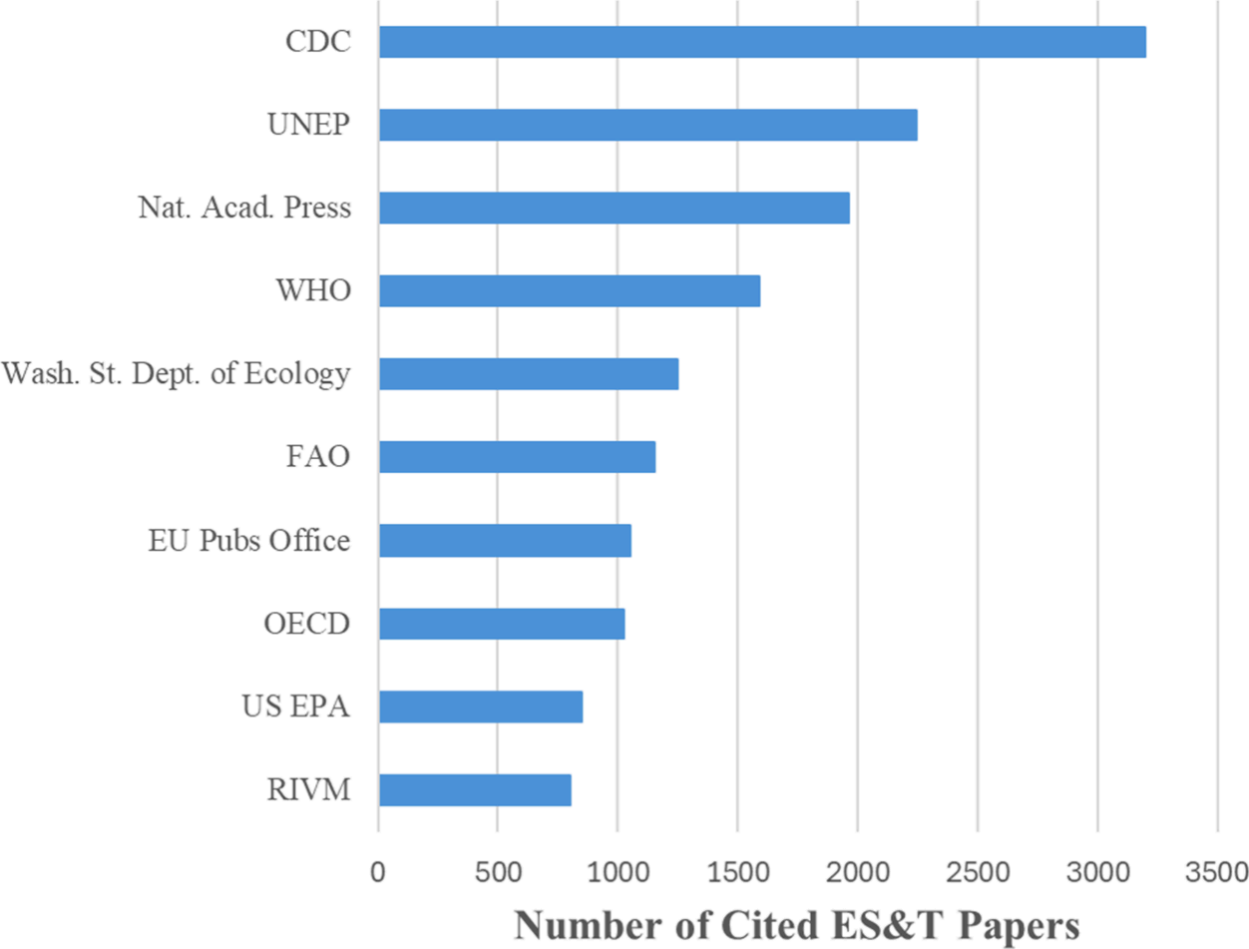
Organizations and citation rate of *ES* *&T* science in policy documents from 1967 to 2025.

The policy impacts of ES&T research are not limited to only U.S. and EU organizations. *ES&T* research has also provided scientific support and evidence to many other countries’ pollution prevention and control for air, water, soil as well as guidance on how to address emerging pollutants. For example, research on critical loading was one basis for the designation of the SO_2_ Pollution Control Zone and Acid Rain Control Zone in 1998 that were implemented globally. Studies on emission reductions during the 2008 Olympic Games in Beijing promoted regional joint air pollution control strategies in China. Recent studies in *ES&T* have been more related to the clean air (PM2.5, ozone, mercury) and carbon neutrality pathways and are impacting policy in China.

There are several reasons that *ES* *&T* has significant policy relevance. The first is that the journal covers a relatively broad scope of environmental topics. There are many papers that are groundbreaking in that they identify the breadth and significance of a previously unknown environmental concern or human health issue, or they provide new methods for analysis or models for toxicity, fate, or exposure assessments, and indicate the best available technologies that are relevant to regulators and policy makers. Papers may also assess the potential impacts of new technologies on the environment and human health before they are broadly introduced. Of equal importance to the topic is the nature and character of the kinds of studies published in *ES* *&T*. *ES* *&T* is renowned for scientific rigor, in large part due to its industry-leading peer review processes that emphasize methodological transparency, reproducibility, environmental relevance, and mechanistic insight.

Some of the most policy relevant works have included, e.g., sources, exposure routes, and toxicological effects of microplastics in the environment; human exposures to PBDEs in house dust or to toxins in electronic cigarettes; high resolution mapping of particulate matter in urban environments to understand equity issues in PM2.5 exposures. *ES* *&T* papers have highlighted the extent of PFAS contamination globally, its ubiquitous presence in breast milk, in wildlife, and its food web impacts, informing regulations on PFAS use and standards for drinking water. Other important topics led by *ES* *&T* research have raised awareness on antimicrobial resistance, endocrine disruptors, and chemical mixture impacts as well as blood lead levels in disadvantaged communities. *ES* *&T* work in systems analysis including lifecycle assessments, the carbon footprint of food distribution (food miles), and the critical need for resource conservation in agricultural systems has all influenced global policies.

*ES&T* research has also led to numerous policy change outcomes that have improved the environment and human health. For example, *ES&T* research documented elevated blood lead levels in infants and toddlers after changes in drinking water disinfection practices in the Washington, DC, area^4^ and demonstrated that partial lead service line replacement often caused short-term spikes in lead, sometimes exceeding prereplacement levels.^5^ These works provided a defensible scientific rationale for policy changes on pipe corrosion control and service line replacements required to protect public health. *ES&T* research and policy analysis also identified early concerns about the impacts of biofuel production on already stressed water resources and on the resulting nutrient loadings.^6–8^ These papers helped to frame the issue (e.g., gallons of water per mile driven) in a way that decision-makers and other stakeholders could interpret. *ES&T* papers documenting the occurrences and concentrations of PFAS chemicals in biota and water systems^9–11^ have been important for setting regulatory limits on specific PFAS compounds in New Jersey and elsewhere. The research published in *ES* *&T* is aimed to be highly relevant to understanding, addressing, and advancing solutions to both current and emerging challenges. The median time for *ES* *&T* papers to be cited in various policy documents is 5 to 6 years from publication. However, of the papers cited in policy documents, about 25% are referenced within the first few years following publication. These papers typically have direct relevance for agencies tackling a high profile and immediate concern (e.g., PFAS, microplastics, and particulate matter). Some foundational *ES* *&T* papers (e.g., those on Great Lakes contaminants and fundamental atmospheric chemistry) continue to be cited in policy documents some 35 years after publication, indicating a lasting impact. For example, the classical paper “Finding Fugacity Feasible” by Mackay is still relevant for policies on POPs.^2^ There are many similar “long-tail” papers with continuing impacts including papers on fate and transport of persistent pollutants (e.g., PCBs, chlorinated solvents, metals, PAHs), mechanistic studies (e.g., metal–organic matter interactions), toxicology and bioaccumulation frameworks (bioavailability and trophic transfer), and some method papers that are still used today (VOC sampling, analytical chemistry). These foundational papers continue to inform new studies, environmental sampling, model development, policy, and technological innovation, especially under frameworks governing PFAS, hazardous air pollutants, contaminated sites, wastewater discharges, and chemical risk assessment. Several of these papers have also been instrumental for education in environmental chemistry and engineering and used for popular textbooks such as “Environmental Organic Chemistry”.^3^

## Future Outlook and Critical Need for Environmental and Human Health Research

The first 60 years of *ES* *&T* have led to and served a prolific and strong global network of environmental and human health researchers. The cutting-edge impactful research articles, reviews, policy analyses, perspectives, viewpoints, and editorials continue to provide new knowledge, data, and solutions needed to keep our planet and its inhabitants healthy and to secure a future environment worth living in.

As we look toward the next decades, new environmental challenges and solutions are constantly emerging that will require this network of global researchers to continue to explore, innovate, and collaborate. For example, identifying sustainable approaches to decarbonize industries and limit anthropogenic CO_2_ emissions will be central to avoiding the most disastrous impacts of climate change. We need to continue to find ways to protect our oceans, agriculture, forests, infrastructure, and communities against stressors like heat, drought, flooding, fire, and disease. We need to better mitigate human exposures to toxic anthropogenic chemicals through the development and scaling of biobased, degradable, benign alternatives. We need to prevent exposure to microplastics, disinfection byproducts, PFAS, and other persistent contaminants.

While these complex, wicked global scale problems seem intractable, the environmental research community has collectively responded to similar challenges in the past, such as managing stratospheric ozone depletion by limiting the use of freon and selected halogenated refrigerants by developing effective alternatives, reducing neurotoxic and reproductive toxic effects of lead by banning lead-containing additives in gasoline and replacing them with less toxic alternatives compatible with catalysts, decreasing the occurrence of massive algal blooms by understanding the role of phosphorus from detergents and then contributing to alternative product formulations, or banning selected persistent organic pollutants (POPs) from commerce based on their demonstrated bioaccumulation and biomagnification while designing safer alternatives. These past environmental wins were made possible through the combined efforts of a global network of scientists, engineers, citizens, policymakers, and educators, a network that *ES* *&T* has helped cultivate for six decades. The same collective effort will be essential to confronting the environmental challenges of the future. We are optimistic that any future environmental challenges can be addressed by this community, and *ES* *&T* will continue to serve to help shape research and action toward a healthier, resilient, and sustainable planet for all in the next 60 years to come.
